# Efficient Brain Age Prediction from 3D MRI Volumes Using 2D Projections

**DOI:** 10.3390/brainsci13091329

**Published:** 2023-09-15

**Authors:** Johan Jönemo, Muhammad Usman Akbar, Robin Kämpe, J. Paul Hamilton, Anders Eklund

**Affiliations:** 1 Division of Medical Informatics, Department of Biomedical Engineering, Linköping University, 581 83 Linköping, Sweden; 2 Center for Medical Image Science and Visualization (CMIV), Linköping University, 581 83 Linköping, Sweden; 3 Center for Social and Affective Neuroscience, Department of Biomedical and Clinical Sciences, Linköping University, 581 83 Linköping, Sweden; 4 Department of Biological and Medical Psychology, University of Bergen, 5020 Bergen, Norway; 5 Division of Statistics and Machine Learning, Department of Computer and Information Science, Linköping University, 581 83 Linköping, Sweden

**Keywords:** brain age, 3D CNN, 2D projections, deep learning

## Abstract

Using 3D CNNs on high-resolution medical volumes is very computationally demanding, especially for large datasets like UK Biobank, which aims to scan 100,000 subjects. Here, we demonstrate that using 2D CNNs on a few 2D projections (representing mean and standard deviation across axial, sagittal and coronal slices) of 3D volumes leads to reasonable test accuracy (mean absolute error of about 3.5 years) when predicting age from brain volumes. Using our approach, one training epoch with 20,324 subjects takes 20–50 s using a single GPU, which is two orders of magnitude faster than a small 3D CNN. This speedup is explained by the fact that 3D brain volumes contain a lot of redundant information, which can be efficiently compressed using 2D projections. These results are important for researchers who do not have access to expensive GPU hardware for 3D CNNs.

## 1. Introduction

Predicting brain age from magnetic resonance imaging (MRI) volumes using deep learning has become a popular research topic recently [1,2,3,4,5,6,7,8,9,10,11,12,13]; see Tanveer et al. [14] for a recent review. More traditional machine learning methods such as regression (often using different features such as the size of different brain regions) have also been used for predicting brain age [15,16,17]. If there is a large difference between the predicted brain age and the biological age of a patient, one can suspect that some disease is present and the difference is therefore an important biomarker [4,18,19]. The motivation behind this is that the brain may age more quickly due to different diseases. Virtually all of the previous deep-learning-based works have used 3D convolutional neural networks (CNNs) to predict brain age, or trained 2D CNNs on all slices in each volume and then combined all the slice predictions for a prediction for the entire volume [2,6,9]. Since 3D CNNs are computationally demanding and require a lot of GPU memory, we therefore propose to instead use 2D projections of the 3D volumes. Compared to previous approaches that use 2D CNNs on volume data [2,6,9], we only use 1–6 images per patient (compared to using all 100–300 slices in a volume).

Using 2D CNNs has many benefits compared to 3D CNNs. For example, 2D CNNs can use cheaper hardware (important for low-income countries), can use networks pre-trained on ImageNet or RadImageNet [20] (there are very few pre-trained 3D CNNs) and in general benefit from the more mature and better optimized 2D CNN ecosystem. They can also have fewer parameters (which can benefit federated learning due to lower bandwith consumption). Furthermore, due to the faster training it is much easier to tune the hyperparameters.

Langner et al. [21] demonstrated that 2D projections of full-body MRI volumes can be used to train 2D CNNs to predict different measures like age. Since brain volumes contain less anatomical variation compared to full-body volumes, it is not clear if the same approach is well suited for brain volumes. Furthermore, Langner et al. only used mean intensity projections, while we also use the standard deviation projections (to better capture the variation between slices).

## 2. Materials and Methods

### 2.1. Data

**Figure 1:**
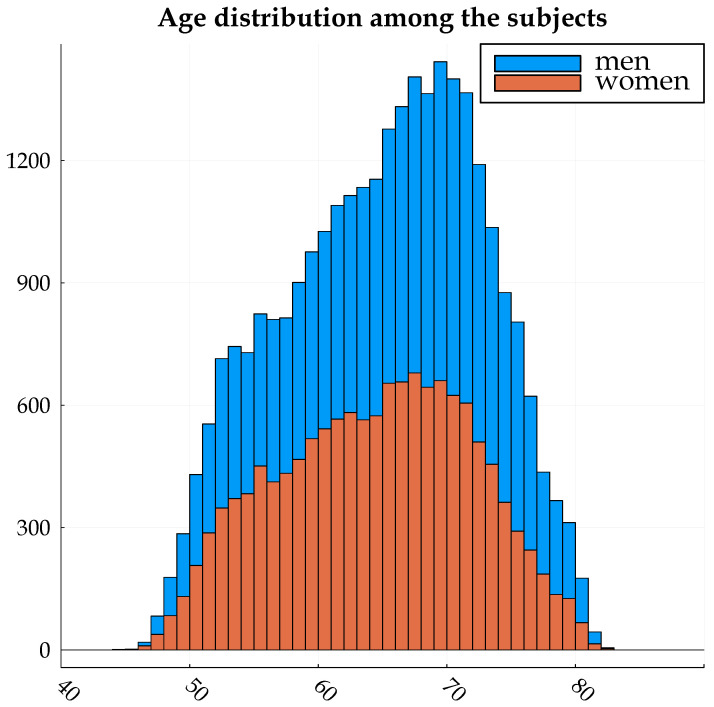
Age distribution for the 29,035 subjects used in this work. The individual bars are further divided to reflect the proportion of each gender within that age group.

**Figure 2:**
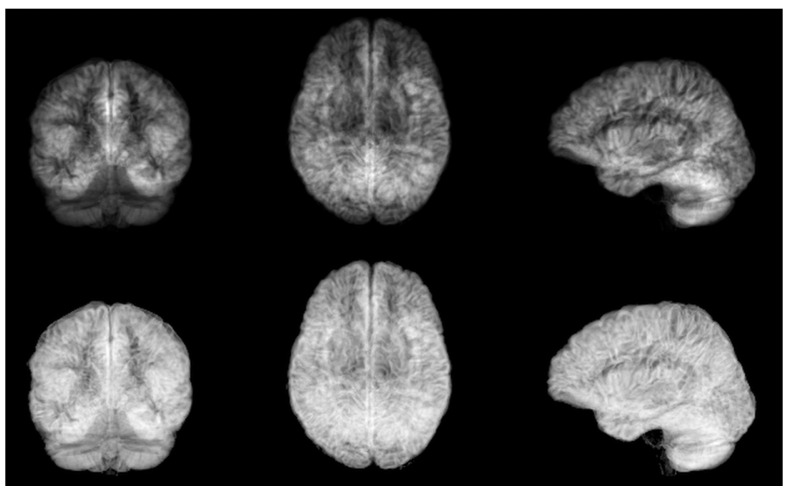
**Top:** mean grey matter likelihood projections on coronal, axial and sagittal planes, for one subject. **Bottom:** standard deviation grey matter likelihood projections on coronal, axial and sagittal planes, for the same subject.

The experiments in this paper are based on T1-weighted brain volumes from 29,035 subjects in UK Biobank [22,23,24]. The age range is 44–82 years with a resolution of 1 year; see Figure 1 for the age distribution. The subjects were divided into 20,324 for training, 4356 for validation and 4355 for testing. FSL FAST [25] was used for each skull-stripped volume, to obtain maps of gray matter (as they have proven to yield better age predictions compared to raw MRI volumes). These gray matter volumes were zeropadded, symmetrically, to match the largest grid (matrix size), resulting in volumes of 256 × 256 × 208 voxels. Each volume was then projected into six 2D images, which represent the mean and standard deviation across axial, sagittal and coronal slices (for one subject at a time). See Figure 2 for the six projections of one subject. The original dataset is about 1.5 TB as 32 bit floats.

### 2.2. Two-Dimensional Projections

**Figure 3:**
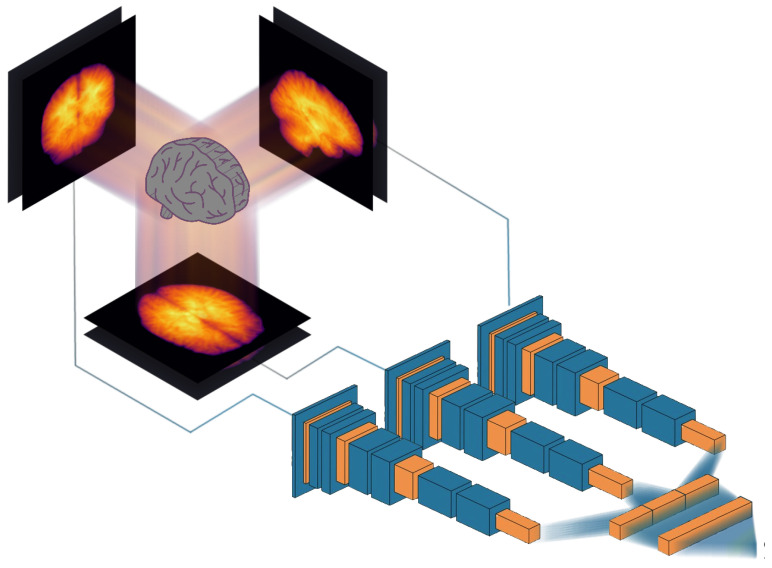
Our proposed approach to obtain efficient brain age prediction using 2D projections of 3D volumes. Each volume is summarized as six 2D images, which represent the mean and standard deviation across axial, sagittal and coronal slices. These 2D images are then fed into three 2D CNNs, and the resulting feature vectors are concatenated and fed into a fully connected layer to predict the brain age.

In this work, we implemented a set of 2D CNNs using the Julia programming language (version 1.6.4) [26] and the Flux machine learning framework (version 0.12.8) [27], wherein the aforementioned projections—typically with two channels each—were fed into their respective stack of convolutional and auxiliary layers (see Figure 3). Instead of training a single multi-channel CNN, three separate CNNs were trained as the important features for sagittal images may be different from the important features for axial images, for example. Each CNN produced 256 features, which were concatenated and fed into a fully connected layer ending in one node with linear output.

The models tested had 13 convolutional layers for each projection (axial, coronal or sagittal). The convolutional stacks had 4 filters in the first layer, which then progressed as the resolution was reduced to 256 filters as mentioned earlier. To explore how some hyperparameters affect the accuracy, the number of convolutional layers was increased to 19 and 25. Furthermore, the number of filters per convolutional layer was also decreased by 50% or increased by 100%. The models had from a little more than 0.8 million to over 8 million trainable parameters.

The training was performed using mean squared error (MSE) as a loss function. Batch normalization and dropout regularization (probability 0.2) were used after every second (or for the models with more layers, third or fourth) convolutional layer, or between the dense layers (probability 0.3 or 0.5). In all cases, the layers follow the order convolution/dense layer → batch normalization → activation → dropout → convolution/dense layer, in accordance with the usage in the articles introducing batch normalization and dropout [28,29]. It has been demonstrated that using dropout and batch normalization together can cause disharmony, but we believe this phenomenon to be alleviated by the layers following the dropout that precede the next batch normalization, especially since these layers always include an increase in the number of features, which Li et al. indicate would be helpful [30]. The dropout rate was arrived at empirically during preliminary tests (not published in this article), which also seems to belie any significant dysergies. Optimization was carried out using the Adam optimizer, with a learning rate of 0.003. Training was always performed for 400 epochs, and the weights were saved every time the validation loss decreased. Furthermore, the training was also performed where the weights of the three 2D CNNs were fixed to be the same (here called iso).

Data augmentation was tentatively explored using the Augmentor module [31], wherein an augmentation pipeline was constructed. The augmented data set consisted of the unaugmented set concatenated with three copies that had been passed through a pipeline of small random pertubations in the form of scaling, shearing, rotation and elastic deformation. This set was randomly shuffled for each epoch of training. As of yet, the code has not successfully been made to work with on-the-fly augmentation, nor have we been able to utilize GPUs for these calculations.

**Figure 4:**
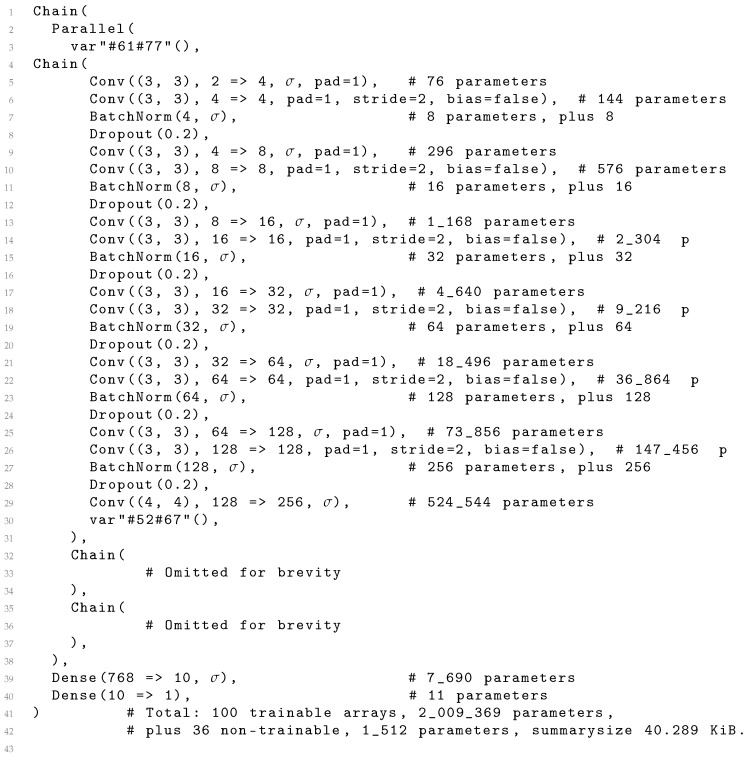
A report on a typical network automatically generated by the Flux framework, expressed as Julia code. Here the Parallel structure holds the three stacks (represented by Chain structures within the Flux framework) of convolutional layers (and some auxiliary layers), which process axial, sagittal and coronal projections. Here, σ denotes the activation function employed after a layer. Because the three stacks are very similar, only the first one is shown. The odd-looking expressions in lines 3 and 30 are anonymous functions used to suitably reformat the data.

Training the networks was performed using an Nvidia (USA) RTX 8000 graphics card with 48 GB of memory. A major benefit of our approach is that all the training images fit in GPU memory (when augmentation was not used), making the training substantially faster since the images did not need to be streamed from the main memory or from the hard drive. One epoch of training with 6 projections from 20,324 subjects took 20–50 s for models with 13 convolution layers per projection (which can be compared to 1 hour for a 3D CNN trained with 12,949 subjects [7]). Our code is available at https://github.com/emojjon/brain-projection-age (accessed on 1 September 2023), and a Julia code for an example network is given in Figure 4.

## 3. Results

Table 1 shows the test prediction accuracies and training times for previously published papers (using 3D CNNs, or 2D CNNs on all slices) and our approach using 2D projections. While several papers used the UK Biobank dataset, the test sets are different, which makes a direct comparison of the test accuracy difficult (we would need to implement and train all other networks on our specific data). Table 2 shows the results from changing the hyperparameters, and when training with fewer subjects. As expected, a smaller training set deteriorates the test accuracy. Increasing the number of filters per layer has a small positive effect, while the effect of increasing the number of convolution layers is not so clear.

**Figure 5:**
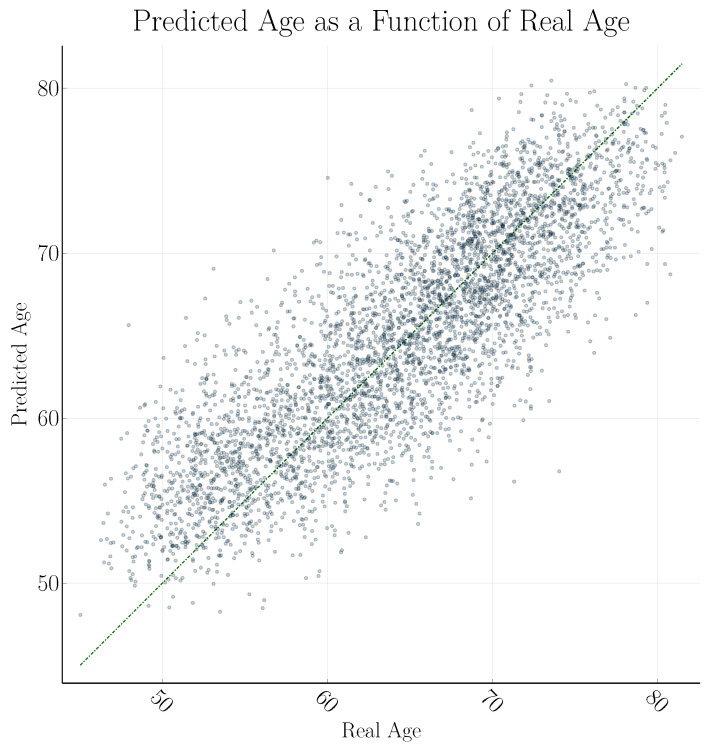
Comparison of real and predicted age in the test set of 4355 subjects, for a model with 19 convolution layers for each projection and using all six channels. The coefficient of determination $r^{2}$ is 0.691.

Our approach is substantially faster compared to previously published papers, even though we are using the largest training set, while our test accuracy is worse. Using the standard deviation to produce 2D projections leads to a slightly higher accuracy, compared to using the mean across slices. Using both mean and standard deviation projections sometimes provides a small improvement, compared to only using the standard deviation. Forcing the three 2D CNNs to use the same weights (referred to as iso) sometimes leads to a higher accuracy, compared to using three independent CNNs. Data augmentation helps to further improve the accuracy, but is currently much slower. To better visualize the relationship between real and predicted age, these are plotted against each other in Figure 5 for an example model.

While several measures could be employed to measure the accuracy of the model, we prefer reporting the mean absolute error on the test set and have also included the root of the mean squared error on the same. This is partly because the former is the most common measure to report in models predicting brain age, and the latter was natural to include because we used the mean squared error as the loss function during training (partly because these measures have the unit years, which we feel make them more intuitive). As an example, the coefficient of determination $r^{2}$ calculated on the test set for the model visualized in Figure 5 is 0.691. It is, however, uncertain to what extent $r^{2}$ lends itself to measure non-linear models such as this.

**Figure 6:**
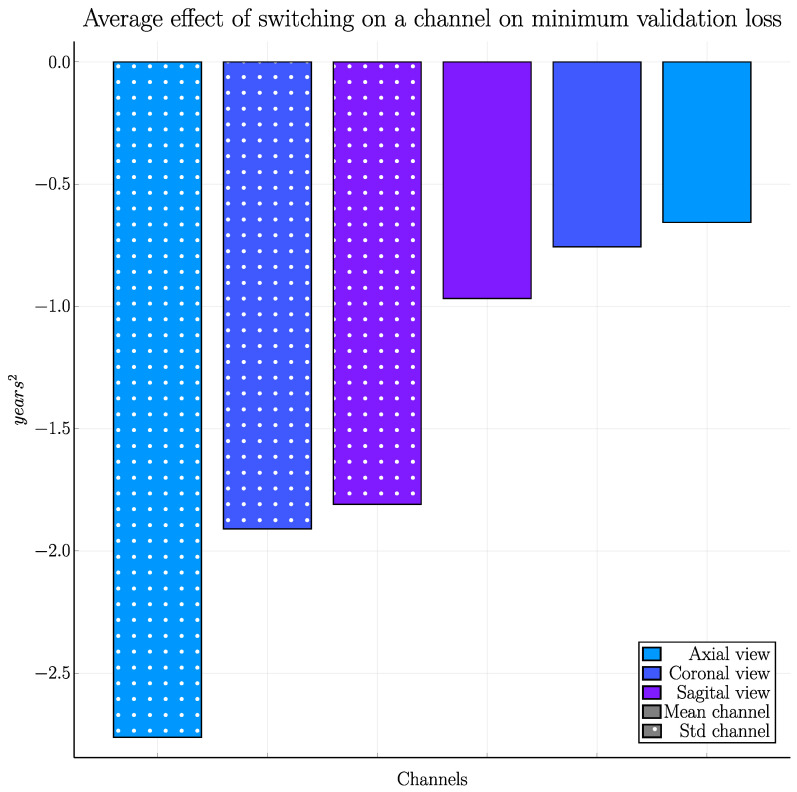
The effect—in the preliminary study on raw intensity volumes—of adding additional channels on the prediction accuracy, averaged over 128 trainings when using different combinations of input channels (64 different input combinations for 2 different learning rates). Adding the standard deviation images (marked with dots in this plot) from the different views has the largest effects and the mean images the smallest.

In a preliminary study, we trained the 2D CNNs repeatedly with 1–6 input projections from the original intensity volumes (the results largely follow the same pattern as grey matter likelihood but with slightly lower accuracy) to see which projections are the most important for the network, resulting in a total of 64 combinations. This was repeated for two learning rates, for a total of 128 trainings. Figure 6 shows the decrease in loss when adding each channel, averaged over said trainings. Clearly, the standard deviation projections are more informative compared to the mean intensity projections.

In the process of training the models, RMSE for both the training set and validation set was observed. While these values are not listed for each model, we noted that for the validation set the values closely follow those for the test set. For the training set, RMSE was typically little more than half that of the test set (at early stopping), indicating some overfitting. As one might expect, this effect became more pronounced as the numbers of trainable parameters grew.

## 4. Discussion

Our results show that our 2D projection approach is substantially faster compared to previous work, although several papers do not report the training time. The speedup will, in our case, not be as large for GPUs with smaller memory, as it is then not possible to put all the training images in the GPU memory (for a preliminary test on a 11 GB card, the training took 3–4 times longer, but this can probably be further optimized). Nevertheless, the possibility to use cheaper hardware is important for many researchers. Compared to other 2D approaches, which use all slices in each volume, our 2D projection approach is substantially faster compared to Huang et al. [2] and Bashyam et al. [6], and our accuracy is also better. Compared to Gupta et al. [9], our approach is faster while our accuracy is lower. Our test accuracy is in general slightly worse compared to 3D CNNs, but our work should rather be seen as a proof of concept. It would be interesting to instead use 2D CNNs pre-trained on ImageNet or RadImageNet [20] as a starting point, instead of training from scratch. However, this option is currently more difficult in Flux compared to other machine learning frameworks. Yet another way to improve test accuracy is to use an ensemble of networks. Using the mean prediction of 5–10 networks will most likely improve the accuracy, while still only requiring about 125–250 min of training.

Although our proposed solution results in a lower accuracy compared to much more time-consuming 3D approaches, an approximate brain age estimate can still be valuable for diagnostic purposes. For example, if a person’s biological age is 35 years and the predicted brain age is 50 years, a slightly lower or higher prediction will still lead to the conclusion that the person’s brain is abnormal.

Langner et al. [21], who used 2D projections of full-body MRI scans (not including the head), obtained a mean absolute error of 2.49 years when training with 23,120 subjects from UK Biobank (training the network took about 8 h). It is difficult to determine if the higher accuracy compared to our work is due to using a VGG16 architecture (pre-trained on ImageNet), or due to the fact that full-body scans contain more information regarding a person’s age, or that the full-body scans in UK Biobank contain separate images representing fat and water. No comparison with a 3D CNN is included in their work.

The demographic in the UK Biobank dataset is relatively homogenous (94.6% of participants were of white ethnicity) and there is evidence of a “healthy volunteer” selection bias [32]. Our 2D projection models are therefore expected to perform less well when applied to data from a more diverse population (e.g., regarding neurological disease, brain size, ethnicity, age). However, this is also true for 3D CNNs trained on UK Biobank data. Whether 2D or 3D CNNs are more affected by a more diverse dataset will be explored in future research.

In future work, we also plan to investigate the effect of adding additional images (channels) that represent the third and fourth moment (skew and kurtosis) across slices, since the results indicate that the standard deviation images are more informative compared to the mean intensity images. Another idea is to use principal component analysis (PCA) across each direction, to instead use eigen slices that represent most of the variance. As can be seen in Table 1, adding more channels will not substantially increase the training time as a higher number of input channels will only affect the first layer of each 2D CNN. This is different from adding more training images to a 2D CNN using each slice in a volume independently, where the training time will increase more or less linearly with more images.

## 5. Conclusions

The conclusion is that using 2D projections from 3D volumes results in large speedups, compared to 3D CNNs. The accuracy is slightly lower with our approach, but we believe that the results can still be used to, for example, detect abnormal brains.

## Figures and Tables

**Table 1:** Comparison of our 2D projection approach and previous publications on brain age prediction (using 3D CNNs, or 2D CNNs on all slices), regarding number of training subjects (N), brain age test accuracy (mean absolute error (MAE) in years, RMSE in parenthesis) and training time. Iso here refers to the fact that the three parallel 2D CNNs (for axial, sagittal and coronal projections) are forced to use the same weights. Even though several publications use the UK Biobank data, a direct comparison of the test accuracy is not possible as different test sets, in terms of size and the specific subjects, were used. The available training times were rescaled to a single GPU, if multi-GPU training was mentioned. The training time for our approach is presented for early stopping, and for the full 400 epochs in parenthesis.

Paper/Settings	Approach	N Subjects	Test Accuracy	Parameters	Training Time
Huang et al., 2017 [2]	2D slices	600	4.00 MAE	-	12 h
Cole et al., 2017 [3]	3D CNN	1601	4.16 MAE	889,960	72–332 h
Wang et al., 2019 [4]	3D CNN	3688	4.45 MAE	-	30 h
Jonsson et al., 2019 [5]	3D CNN	809	3.39 MAE	-	48 h
Bashyam et al., 2020 [6]	2D slices	9383	3.70 MAE	-	10 h
Peng et al., 2021 [7]	3D CNN	12,949	2.14 MAE	3 million	130 h
Bellantuono et al., 2021 [8]	Dense	800	2.19 MAE	-	-
Gupta et al., 2021 [9]	2D slices	7312	2.82 MAE	998,625	6.75 h
Ning et al., 2021 [10]	3D CNN	13,598	2.70 MAE	-	96 h
Dinsdale et al., 2021 [11]	3D CNN	12,802	2.90 MAE	-	-
Lee et al., 2022 [12]	3D CNN	1805	3.49 MAE	70,183,073	24 h
Dropout between conv					
0.2 dropout rate					
Ours, 3 mean channels	2D proj	20,324	3.55 (4.49)	2,009,261	22 min (3 h 53 min)
Ours, 3 std channels	2D proj	20,324	3.51 (4.43)	2,009,261	24 min (3 h 30 min)
Ours, all 6 channels	2D proj	20,324	3.53 (4.44)	2,009,369	24 min (3 h 26 min)
Ours, all 6 channels, iso	2D proj	20,324	3.46 (4.38)	827,841	25 min (4 h 36 min)
Dropout between dense					
0.3 dropout rate					
Ours, 3 mean channels	2D proj	20,324	3.70 (4.66)	2,009,261	22 min (3 h 12 min)
Ours, 3 std channels	2D proj	20,324	3.67 (4.62)	2,009,261	27 min (4 h 27 min)
Ours, all 6 channels	2D proj	20,324	3.56 (4.47)	2,009,369	27 min (3 h 32 min)
Ours, all 6 channels, iso	2D proj	20,324	3.63 (4.56)	827,841	28 min (4 h 23 min)
Dropout between conv					
0.2 dropout rate					
trained with augmentation					
Ours, 3 mean channels	2D proj	20,324 ^1^	3.44 (4.31)	2,009,261	> 3 days ^2^
Ours, 3 std channels	2D proj	20,324 ^1^	3.40 (4.33)	2,009,261	> 3 days ^2^
Ours, all 6 channels	2D proj	20,324 ^1^	3.47 (4.40)	2,009,369	> 3 days ^2^
Ours, all 6 channels, iso	2D proj	20,324 ^1^	3.85 (4.80)	827,841	> 3 days ^2^

^1^ The model is trained with an augmented set of 20,324 + 60,972 = 81,296 pseudo subjects, but all are derived from the original 20,324 subjects. ^2^ This was a preliminary exploration of whether augmentation was motivated. For more competitive speeds, further optimisation is required

**Table 2:** Here, we show variations of other aspects of the model in order to evaluate their effect. All modifications are relative to the models in the second section of Table 1. The training time for our approach is presented for early stopping, and for the full 400 epochs in parentheses.

Settings	Approach	N Subjects	Test Accuracy	Parameters	Training Time
Dropout between conv					
0.2 dropout rate					
trained using only					
2000 subjects					
Ours, 3 mean channels	2D proj	2000	4.05 (5.09)	2,009,261	18 min (22 min)
Ours, 3 std channels	2D proj	2000	4.01 (5.08)	2,009,261	20 min (22 min)
Ours, all 6 channels	2D proj	2000	4.06 (5.13)	2,009,369	7 min (22 min)
Ours, all 6 channels, iso	2D proj	2000	4.13 (5.18)	827,841	8 min (27 min)
Dropout between conv					
0.2 dropout rate					
trained using only					
6376 subjects					
Ours, 3 mean channels	2D proj	6376	3.75 (4.74)	2,009,261	7 min (58 min)
Ours, 3 std channels	2D proj	6376	3.73 (4.72)	2,009,261	4 min (58 min)
Ours, all 6 channels	2D proj	6376	3.73 (4.73)	2,009,369	50 min (1 h 7 min)
Ours, all 6 channels, iso	2D proj	6376	3.77 (4.75)	827,841	53 min (1 h 16 min)
Dropout between conv					
0.2 dropout rate					
half as many filters					
Ours, 3 mean channels	2D proj	20,324	3.61 (4.51)	505,037	37 min (2 h 40 min)
Ours, 3 std channels	2D proj	20,324	3.61 (4.57)	505,037	43 min (3 h 3 min)
Ours, all 6 channels	2D proj	20,324	3.49 (4.40)	505,091	17 min (3 h 10 min)
Ours, all 6 channels, iso	2D proj	20,324	3.49 (4.39)	209,167	40 min (4 h 52 min)
Dropout between conv					
0.2 dropout rate					
twice as many filters					
Ours, 3 mean channels	2D proj	20,324	3.45 (4.39)	8,015,333	25 min (4 h 51 min)
Ours, 3 std channels	2D proj	20,324	3.45 (4.37)	8,015,333	23 min (4 h 52 min)
Ours, all 6 channels	2D proj	20,324	3.40 (4.30)	8,015,549	23 min (4 h 55 min)
Ours, all 6 channels, iso	2D proj	20,324	3.42 (4.33)	3,293,773	19 min (5 h 39 min)
Dropout between conv					
0.2 dropout rate					
with 19 convolution layers					
per stack rather than 13					
Ours, 3 mean channels	2D proj	20,324	3.56 (4.50)	2,599,697	37 min (4 h 24 min)
Ours, 3 std channels	2D proj	20,324	3.49 (4.40)	2,599,697	50 min (4 h 39 min)
Ours, all 6 channels	2D proj	20,324	3.40 (4.28)	2,599,805	31 min (4 h 43 min)
Ours, all 6 channels, iso	2D proj	20,324	3.37 (4.26)	1,024,653	60 min (5 h 44 min)
Dropout between conv					
0.2 dropout rate					
with 25 convolution layers					
per stack rather than 13					
Ours, 3 mean channels	2D proj	20,324	3.49 (4.41)	3,189,985	1 h 22 min (5 h 29 min)
Ours, 3 std channels	2D proj	20,324	3.47 (4.38)	3,189,985	1 h 20 min (5 h 27 min)
Ours, all 6 channels	2D proj	20,324	3.50 (4.47)	3,190,093	1 h 37 min (5 h 46 min)
Ours, all 6 channels, iso	2D proj	20,324	3.48 (4.38)	1,221,465	1 h 14 min (7 h 26 min)

## Data Availability

The data used in this work are available through UK Biobank, https://www.ukbiobank.ac.uk/.
